# Cephalosporin-Resistant *Neisseria gonorrhoeae* Clone, China

**DOI:** 10.3201/eid2404.171817

**Published:** 2018-04

**Authors:** Shao-Chun Chen, Yue-Ping Yin, Xiang-Sheng Chen

**Affiliations:** Chinese Academy of Medical Sciences and Peking Union Medical College, Nanjing, China (S.-C. Chen, Y.-P. Yin, X.-S. Chen);; Chinese Center for Disease Control and Prevention, Nanjing (S.-C. Chen, Y.-P. Yin, X.-S. Chen)

**Keywords:** *Neisseria gonorrhoeae*, antimicrobial resistance, internationally spread clone, *N. gonorrhoeae* multiantigen sequence type, NG-MAST G1407, multilocus sequence type, MLST 1901, extended-spectrum cephalosporin, sexual transmitted infections, bacteria, China, gonorrhea

## Abstract

Cephalosporin-resistant *Neisseria gonorrhoeae* is a major public health concern. *N. gonorrhoeae* of multiantigen sequence type G1407 and multilocus sequence type 1901 is an internationally spreading cephalosporin-resistant clone. We detected 4 cases of infection with this clone in China and analyzed resistance determinants by using *N. gonorrhoeae* sequence typing for antimicrobial resistance.

Gonorrhea, the second most prevalent sexually transmitted infection (STI) globally, remains a major public health concern in China. From 2015 to 2016, the reported cases of gonorrhea in China increased by 14.7% (100,245 to 115,024) (*1*). The extended-spectrum cephalosporin ceftriaxone has been recommended as monotherapy to treat gonorrhea in China since 2007 (*2*), but resistance to this drug emerged almost at the same time (*3*). Presently, the transmission of internationally spread cephalosporin-resistant clones in China has become a threat to effectively controlling gonorrhea (*4*). Strains with *N. gonorrhoeae* multiantigen sequence type (NG-MAST) G1407 and multilocus sequence type (MLST) 1901 have been successful clones associated with cephalosporin resistance and have caused clinical treatment failures in France and Spain (*5*,*6*); these strains have also became the predominant clones in the United Kingdom (*7*) and Japan (*8*) and among US men who have sex with men (*9*). Here we report 4 cephalosporin-resistant NG-MAST G1407/MLST 1901 clones identified out of 2,038 isolates collected through China’s Gonococcal Resistance Surveillance Program during 2015–2016.

Demographic and clinical information for the 4 case-patients are summarized in Technical Appendix Table 1. All case-patients were adult men; gonococcal isolates were obtained from urethral swab samples. The 4 men had obvious urethral discharge and were diagnosed with acute urethritis. Gram staining and culture of the urethral swabs were positive for gonococcal infection. One of the 4 patients self-reported being a man who has sex with men. One of the infections, occurring in Zhejiang Province, was treated with a single-dose regimen of spectinomycin (4 g); the other 3 infections, occurring in the municipality of Chongqing, were treated with a 2-dose regimen of ceftriaxone (1 g) administered over 2 days. Test-of-cure follow-ups were not performed.

All strains were transferred to the reference laboratory at the National Center for Sexually Transmitted Disease Control, Chinese Center for Disease Control and Prevention. Gram staining, a rapid oxidase reaction test, and a carbohydrate utilization test confirmed the identification of *N. gonorrhoeae*. We determined antimicrobial susceptibility to ceftriaxone (CRO), cefixime (CFM), spectinomycin (SPT), azithromycin (AZM), ciprofloxacin (CIP), and penicillin (PEN) by using the agar dilution method. We detected β-lactamase (penicillinase)–producing *N. gonorrhoeae* isolates by using a nitrocefin solution filter paper test. These strains were resistant to CRO, CFM, PEN, and CIP but susceptible to AZM and SPT based on susceptibility and resistance breakpoints from the European Committee on Antimicrobial Susceptibility Testing (http://www.eucast.org/clinical_breakpoints) (Table). MICs of ceftriaxone ranged from 0.25 to 0.50 mg/L, and MICs of cefixime ranged from 0.5 to 1.0 mg/L.

**Table:** MICs of antimicrobial drugs for *Neisseria gonorrhoeae* isolates from 4 case-patients with cephalosporin-resistant NG-MAST G1407/MLST 1901 infections identified through the national Gonococcal Resistance Surveillance Program, China, 2015–2016*

Case-patient no.	MIC, mg/L						
	CRO	CFM	CIP	PEN	SPT	AZM	PPNG
1	0.5/R	0.5/R	8/R	16/R	16/S	1/S	No
2	0.5/R	1/R	32/R	16/R	32/S	0.5/S	No
3	0.5/R	0.5/R	32/R	16/R	32/S	1/S	No
4	0.25/R	0.5/R	32/R	16/R	64/S	1/S	No

*AZM, azithromycin; CFM, cefixime; CIP, ciprofloxacin; CRO, ceftriaxone; MLST, multilocus sequence type; NG-MAST, *N. gonorrhoeae* multiantigen sequence type; PEN, penicillin; PPNG, penicillinase-producing *N. gonorrhoeae*; R, resistant; S, susceptible; SPT, spectinomycin.

We performed NG-MAST and MLST genotyping to identify the sequence types (*10*). MLST showed all 4 strains to be type 1901, and NG-MAST showed the Zhejiang strain to be sequence type (ST) 10332 and the Chongqing strains to be ST1407. ST10332 has a 2-basepair difference in the *porB* (*porB*6067) gene from that of ST1407 (*porB*908) and belongs to genogroup G1407. We used *N. gonorrhoeae* sequence typing for antimicrobial resistance (NG-STAR) to identify the characteristics of resistance determinants (*11*). NG-STAR showed 2 of the Chongqing strains to be ST90; the third Chongqing strain was ST194. The strain isolated in Zhejiang was ST507. All 4 strains had type XXXIV mosaic *penA* (*penA* 34.001), −35A Del in the *mtrR* promoter (*mtrR*1), G120K-A121N/D in PorB (PorB8/11), L421P in PonA (PonA1), S91F-D95A/G in GyrA (GyrA1/7), S87R in ParC (ParC3), and wild-type 23srRNA (23 srRNA0) (Technical Appendix Table 2).

We conclude that the internationally reported cephalosporin-resistant NG-MAST G1407/MLST 1901 *N. gonorrhoeae* clone has spread into China. Genotyping and resistance determinants analysis showed similarity to the predominant G1407/MLST 1901 clone reported in other regions (*7*–*9*), indicating that importation into and transmission within China has occurred. Our findings suggest that increased monitoring of this clone by China’s Gonococcal Resistance Surveillance Program will be vital for monitoring trends in antimicrobial resistance.

Technical Appendix Demographic and clinical information for 4 case-patients with cephalosporin-resistant NG-MAST G1407/MLST 1901 gonococcal infections identified through China’s Gonococcal Resistance Surveillance Program during 2015–2016, and results of isolate genotyping and resistance determinants analysis.
